# Temperature and Diseases of the Year 1842

**Published:** 1843-01

**Authors:** 


### TEMPERATURE AND DISEASES OF THE YEAR 1842.

Now that the calendar year 1842 has ended, we may affirm that it presented extremes between winter and summer, of much less than the usual extent; for while the former was one of the warmest, the latter was among the coldest we ever experience. This is true, we believe, of all parts of the west, from the Delta of the Mississippi, to the shores of Lake Superior. We may quote from Professor Loomis' Reports, that November and December, 1841, and January, February, March, and April of 1842, all had a mean heat *above* what belongs to them; while May, June, July, and August, were *below* their usual mean; finally, September was above it. Frost occurred in each summer month at Hudson, the place of his observations, N. lat. 41° 14′ 40″, at an elevation of 1112 feet above the Ocean. Thus the climate of the west, during that year, so far as temperature was concerned, was much more assimilated to that of Europe, than we usually have it. Now, the phlegmasiæ of last winter, were as frequent and violent as usual; but from all that has reached us, the diseases of summer and autumn were few and mild. We know from personal observation, that the Lake shores were almost as healthy as the banks of mountain rivulets, having traversed them extensively in July, August, and September, within latitudes usually infested with summer and autumnal fevers; and, if not "finding none," scarcely meeting with specimens enough to present their characteristic features. The valley of the Ohio, was almost as free, and in the South there seems to have been an equal comparative exemption.

Here, then, is a cotemporaneous low temperature of summer and autumn, and a prevailing abatement of the epidemics of those seasons? Do these agreeable phenomena stand in the relation of cause and effect?

We beg our readers, every where, to favour us with their observations on the heat, weather, and diseases of the year, to which these remarks relate; that we may bring them together, and see what conclusion will arise (or precipitate) from the play of their affinities.

